# Availability and reimbursement of fixed-dose combination medicines for hypertension treatment in Central and Eastern Europe

**DOI:** 10.3389/fphar.2026.1889485

**Published:** 2026-10-08

**Authors:** Rafał Nowak, Przemysław Holko, Iris Hoxha, Alexandra Savova, Pero Draganić, Jana Skoupa, Agnes Männik, Zoltán Kaló, Arturs Praskilevics, Kristina Garuoliene, Ljubica Shuturkova, Elena Chitan, Natasa Duborija-Kovacevic, Michał Owca, Ioana Bianchi-Manaila, Nikola Stefanovic, Tomas Tesar, Tamás Ágh, Paweł Kawalec

**Affiliations:** 1 Department of Nutrition and Drug Research, Institute of Public Health, Faculty of Health Science, Jagiellonian University Medical College, Kraków, Poland; 2 Department of Pharmacy, Faculty of Medicine, University of Medicine, Tirana, Albania; 3 Department “Organization and Economics of Pharmacy”, Faculty of Pharmacy, Medical University of Sofia, Sofia, Bulgaria; 4 National Council on Prices and Reimbursement of Medicinal Products, Sofia, Bulgaria; 5 Agency for Medicinal Products and Medical Devices of Croatia, Zagreb, Croatia; 6 Faculty of Biotechnology and Drug Development, University of Rijeka, Rijeka, Croatia; 7 EconHealth s.r.o., Prague, Czechia; 8 Institute of Family Medicine and Public Health, University of Tartu, Tartu, Estonia; 9 Center for Health Technology Assessment, Semmelweis University, Budapest, Hungary; 10 Syreon Research Institute, Budapest, Hungary; 11 Department of Pharmaceutical Chemistry, Faculty of Pharmacy, Riga Stradins University, Riga, Latvia; 12 Red Cross Medical College of Riga Stradins University, Riga, Latvia; 13 Pharmacy and Pharmacology Center, Institute of Biomedical Sciences, Faculty of Medicine, Vilnius University, Vilnius, Lithuania; 14 Faculty of Pharmacy, Ss. Cyril and Methodius University in Skopje, Skopje, North Macedonia; 15 Department of Social Pharmacy, Nicolae Testemitanu State University of Medicine and Pharmacy, Chişinău, Moldova; 16 Department of Pharmacology and Clinical Pharmacology, Faculty of Medicine, University of Montenegro, Podgorica, Montenegro; 17 Romanian Association of International Medicine Manufacturers (ARPIM), Bucharest, Romania; 18 Department of Pharmacy, Faculty of Medicine, University of Niš, Niš, Serbia; 19 Department of Organisation and Management in Pharmacy, Faculty of Pharmacy, Comenius University in Bratislava, Bratislava, Slovakia; 20 Medication Adherence Research Group, Center for Health Technology Assessment and Pharmacoeconomic Research, University of Pécs, Pécs, Hungary

**Keywords:** FDC, fixed-dose combination, hypertension, medicine availability, reimbursement policy

## Abstract

**Introduction:**

Fixed-dose combinations (FDCs) represent an important strategy in modern pharmacotherapy and are particularly beneficial in the treatment of chronic conditions such as hypertension because they reduce pill burden and may improve patient adherence to therapy.

**Objectives:**

The primary objective of this study was to compare reimbursement, pricing, and availability policies for FDC medicines used in the treatment of arterial hypertension across 15 CEE countries. Additionally, the study aimed to assess differences in HTA requirements and patient co-payments among the analyzed countries.

**Methods:**

Data were collected from the reimbursement systems of 15 CEE countries using a questionnaire distributed to native experts in each country to ensure contextual accuracy. The list of potential active substance combinations was derived from established clinical guidelines. Associations were assessed using Spearman’s rank correlation coefficients and generalized linear mixed models (GLMMs).

**Results:**

Substantial variation in reimbursement requirements was observed across CEE countries. The greatest variability concerned the inclusion of adherence and persistence outcomes in reimbursement decision-making processes. Seven countries considered these aspects in their reimbursement assessments, whereas the remaining countries did not. The four most frequently reimbursed two-component FDCs were combinations of ARBs with CCBs, ACEIs with CCBs, ACEIs with diuretics, and ARBs with diuretics. Public pharmaceutical expenditure related to hypertension treatment varied substantially across countries; with Estonia allocating the highest proportion of hypertension reimbursement expenditure to FDCs (68% in 2022). The time required for inclusion on reimbursement lists also differed markedly, ranging from less than 3 months in Latvia and Lithuania to more than 12 months in Albania and Romania. Spearman correlation analyses demonstrated that the total number of reimbursed FDCs in a given country was significantly associated with HTA requirements for FDCs containing previously reimbursed components (ρ = 0.641, p = 0.011) and with the time from reimbursement application to inclusion on the reimbursement list (ρ = −0.548, p = 0.041). Multivariable GLMM analysis confirmed that: i) countries with less stringent requirements for FDCs containing already reimbursed components (i.e., countries that either do not require an HTA assessment or make the HTA requirement contingent on the manufacturer’s proposed price) had a significantly higher number of reimbursed FDCs (p = 0.001); and ii) countries with less stringent HTA requirements for FDCs (i.e., those that do not require additional comparators beyond the individual FDC components) also tended to have a higher number of reimbursed FDCs (p = 0.010).

**Conclusion:**

Across CEE countries, less burdensome HTA and reimbursement requirements for FDCs composed of already known and reimbursed active substances were associated with broader reimbursement of single-pill antihypertensive therapies. Streamlining assessment pathways for well-justified, guideline-supported combinations could improve patient access and better align reimbursement practice with current clinical guidelines, while preserving evidence-based decision-making. Common EU-level clinical assessment processes may further support consistency across national HTA systems, although pricing and reimbursement decisions remain within national competence.

## Highlights

- Across 15 Central and Eastern European countries, the rules for approving and paying for single-pill (fixed-dose) combination medicines for hypertension differ a lot, and this translates into big differences in how many such medicines patients can actually access—and how quickly they become available.
- Countries that use simpler, less burdensome reimbursement evaluations—especially when the combination is made from ingredients that are already reimbursed—tend to reimburse more of these single-pill combinations.
- This suggests that streamlining reimbursement requirements for these “already-known ingredient” combinations could improve patient access and better match current clinical recommendations that favor single-pill combinations for many patients.

## 1 Introduction

Fixed-dose combinations (FDCs), which involve the co-formulation of two or more active pharmaceutical ingredients in a single dosage form, have become a pivotal strategy in modern pharmacotherapy. This approach is particularly valuable for managing chronic and complex diseases, such as hypertension, diabetes, HIV/AIDS, and tuberculosis, which often require more than one medication to achieve optimal therapeutic outcomes (Kim and Weon, 2021). Orally administered FDCs, also referred to as single-pill combinations, are typically formulated as capsules or tablets containing two or more active substances. This form of pharmacotherapy reduces pill burden and is recommended to improve patient adherence (Collier, 2012; McEvoy et al., 2024; Wei et al., 2023). Consequently, this approach can enhance treatment effectiveness and mitigate the severity of side effects (DiPette et al., 2019). The highest concentration of FDC launches across therapeutic areas has been observed in the cardiovascular domain (IQVIA, 2023).

Hypertension is one of the conditions commonly managed with FDCs. It is defined by the World Health Organization (WHO) as a sustained blood pressure exceeding 140/90 mmHg. If left untreated, hypertension can lead to serious consequences, such as premature death (WHO, 2025a). The global prevalence of hypertension among adults is approximately 33%, compared to 37% in Europe (WHO, 2023). Within the European countries, prevalence varies considerably, ranging from 22% in Switzerland to 49% in Poland (WHO, 2025b).

Despite decades of advancements in diagnosis and treatment, alarming statistics reveal that 46% of adults with hypertension remain unaware of their condition. Among those receiving treatment, only 21% achieve their therapeutic targets (Saklayen and Deshpande, 2016; WHO, 2025a). According to Eurostat, treatable mortality rates from hypertensive diseases in Europe rose from 3.5 per 100,000 in 2019 to 4.57 per 100,000 in 2021 (Eurostat, 2024). This indicator refers to deaths that could have been prevented through timely and effective treatment or other healthcare interventions (OECD, 2023).

The therapeutic approach to hypertension comprises two main components: lifestyle modification—including diet and physical activity—and pharmacotherapy. The effectiveness of lifestyle changes largely depends on an individual’s ability to adopt and maintain behavioral changes, while the success of pharmacotherapy initially relies on the appropriate prescription of medications. According to the WHO’s *Global Report on Hypertension* published in 2023, antihypertensive drugs are among the most critical elements of hypertension control programs (WHO, 2023). The decision to initiate pharmacotherapy, as well as the selection and administration of treatment, is made by the treating physician. A wide range of medicinal products with different mechanisms of action is available for the treatment of hypertension. The five major classes of antihypertensive medications include angiotensin receptor blockers (ARBs), angiotensin-converting enzyme inhibitors (ACEIs), calcium channel blockers (CCBs), thiazide and thiazide-like diuretics, and beta-blockers (Wei et al., 2023).

The selection of appropriate pharmacological agents or therapeutic regimens can be complex and should be informed by the most up-to-date clinical guidelines, alongside considerations of drug availability and patient access. Both the 2023 European Society of Hypertension (ESH) Guidelines and the 2024 ESC Guidelines for the Management of Elevated Blood Pressure and Hypertension recommend the use of combination antihypertensive therapy, preferably as single-pill combinations, for most patients requiring pharmacological treatment (Mancia et al., 2023; McEvoy et al., 2024). Clinical guidelines evolve over time in response to emerging high-quality evidence from research. These updates may involve changes in the preferred medications, treatment regimens, or the adoption of new combinations of active ingredients.

Selecting an appropriate pharmacological strategy is only one aspect of successful hypertension treatment. The prescribed medications must also be available on the market in the patient’s country. Timely registration of medicines remains a challenge and plays a critical role in establishing a robust and reliable supply chain, which is an especially important consideration for FDCs used in hypertension management (WHO, 2025a). Another potential barrier is the cost of medication. Antihypertensive products must be affordable for the average patient or supported by a reimbursement system to ensure access.

Medicines considered for inclusion on a reimbursement list are often evaluated through a health technology assessment (HTA) process, which supports evidence-based reimbursement decisions. A reimbursement dossier may include several HTA components. The clinical analysis provides evidence on the therapeutic benefits of the medicine, based on all available clinical data. The economic analysis applies pharmacoeconomic methods, aligned with the clinical findings. Additionally, a budget impact analysis (BIA) may be conducted to estimate the financial implications of reimbursing the medicine within a specific healthcare setting. The use of HTA components can vary across countries (Mela et al., 2023). Consequently, reimbursement decisions for the same medicinal product may also differ from country to country. These discrepancies may be attributed to variations in HTA processes, pricing negotiations with manufacturers, national reimbursement systems, and the size of the available reimbursement budget. According to the literature, FDCs can be a cost-effective option (Jowett et al., 2017; Bryan et al., 2023; Levy et al., 2024).

The therapeutic and economic considerations discussed above are particularly important given that hypertension pharmacotherapy is typically chronic and lifelong (Wei et al., 2023). In low- and middle-income countries, combination antihypertensive medications account for a substantial share (45.5%) of total antihypertensive use, surpassing non-combination formulations (Jayawardana et al., 2024). In Central and Eastern European (CEE) countries, variability in healthcare policies, economic constraints, and regulatory environments can potentially impact the accessibility and affordability of FDCs. Reimbursement practices play a critical role in determining whether patients can access and benefit from these innovative therapies. The rising use of FDCs for hypertension in Bulgaria highlights the importance of evaluating reimbursement policies across the CEE countries. Notably, 13 of the top 20 countries (out of 75 analyzed) with the highest antihypertensive drug consumption—measured in standard units per 1,000 inhabitants per day—are located in the CEE region (Jayawardana et al., 2024; Mitkova et al., 2016). Despite this, the current state of FDC reimbursement for hypertension, including regional policy differences, has not been comprehensively reviewed. Moreover, adequate data on the challenges and opportunities associated with this class of medicinal products are lacking.

This is the first comprehensive comparative analysis of HTA and reimbursement requirements for antihypertensive FDCs across CEE countries. The aim of the study was to compare the reimbursement, pricing policies as well as availability for FDC medicines used in the treatment of arterial hypertension across CEE countries, including Albania, Bulgaria, Croatia, the Czech Republic, Estonia, Hungary, Latvia, Lithuania, North Macedonia, the Republic of Moldova, Montenegro, Poland, Romania, Serbia, and Slovakia. Additionally, the study sought to provide insights into potential differences in approaches to the HTA process and in patient co-payments.

## 2 Methods

### 2.1 Study design and questionnaire

The study was designed to collect specific data from the reimbursement systems of CEE countries regarding FDCs used in hypertension treatment. The most recent regulations, detailed procedures, and official documents—when publicly available on regulatory authorities’ websites—are typically published in the respective national languages. This poses challenges in accurately reading, interpreting, and translating the information. To ensure contextual accuracy and a clear understanding of local practices, a questionnaire was developed and distributed to country-specific native experts.

The questionnaire was developed to document the process of incorporating FDCs used for hypertension into national reimbursement schemes. The questionnaire comprises four main domains. The introduction and respondent details section explains the study objectives and collects personal and professional information to ensure transparency and voluntary participation. The reimbursement and pricing domain explores national regulations, application procedures, health technology assessment requirements, economic analyses, price negotiations, and patient co-payment rules to identify differences between FDCs and single-component medicines. The FDC availability section evaluates accessibility, implementation of international treatment guidelines, and lists reimbursed combinations of active substances, while also identifying barriers to use. Finally, the financial and reference data domain requests public spending figures for medicines and FDCs and provides space for sources, figures, and links to official reimbursement lists. The list of possible active substance combinations was derived from the 2023 European Society of Hypertension Guidelines. Ten possible combinations were identified and included in the questionnaire, with the option to include additional combinations not listed. The FDCs were classified based on their active substance combinations, which include: ARBs with CCBs, ACEIs with diuretics, ARBs with diuretics, ACEIs with CCBs, CCBs with diuretics, ACEIs with beta-blockers, CCBs with beta-blockers, and diuretics with beta-blockers. Most questions were multiple-choice, and the design encouraged respondents to provide clarifications and additional comments. The selection of appropriate national experts was based on three primary sources: 1) peer-reviewed publications in the fields of reimbursement policy and market access (identified through searches in PubMed, Embase, and Google Scholar); 2) university websites featuring relevant research; and 3) regulatory authority websites. The questionnaire was sent to all 18 CEE countries in which appropriate experts were identified. In the case of Slovenia, Bosnia, and Ukraine, email invitations to participate in the study were either denied or remained unanswered. The procedure involved sending a second invitation email 2 weeks after the first if there was no response. A total of 34 experts were invited. The questionnaires were returned from April until the end of 2024. Supplementary Table S6 shows the characteristics of experts.

### 2.2 Data management

After data collection, a descriptive analysis was performed to identify similarities and differences among countries in the areas covered by the questionnaire.

The total number of reimbursed FDCs for each country was extracted from the questionnaires. Each extracted chemical and dosage combination was checked in the databases provided by the experts. The assignment of specific combinations to specific FDC groups was verified.

The questionnaire was designed to allow for internal validation (please see Supplementary Appendix). Questions concerning one aspect were later expanded upon to check the consistency of the answers. If an inconsistency was observed, direct contact was made with the expert who prepared the questionnaire to resolve it. After collecting all questionnaires and resolving inconsistencies, a first draft of the manuscript was sent to all experts for approval and comment. The follow-up process was limited to factual reconciliation of reported data and did not alter the substantive meaning of a response from each country. Inconsistencies occurred due to misunderstandings of the questions.

The cost obtained from questionnaires was converted to Purchasing Power Standards (PPS), which is an artificial currency unit with equal purchasing power across 27 EU countries. PPS was calculated by dividing value in national currency by Purchasing Power Parities (PPPs) with EU27_2020 = 1. PPPs with EU27_2020 = 1 are currency conversion rates published by Eurostat (the EU’s statistical office) to adjust for price level differences across countries. The notation EU27_2020 = 1 means the European Union (27 member states from 2020 onward, excluding the United Kingdom) serves as the reference base, set to 1. Values of PPP for each country and year were taken from Eurostat database (Eurostat, 2025). Additionally, the share of FDCs in hypertension-related reimbursement spending was calculated as the proportion of total public reimbursement expenditure on hypertension medicines allocated to FDCs in a given country. No imputation was performed, and missing data was reported as (not available).

### 2.3 Statistical analysis

All study results were summarized as frequencies (categorical variables). Unadjusted differences between countries were assessed with Pearson’s χ^2^ test. Monotonic, unadjusted associations between the total number of reimbursed FDCs and prespecified questionnaire domains were examined using Spearman’s rank correlation coefficient (ρ). To identify aspects associated with the total number of reimbursed FDCs, generalized linear mixed models (GLMMs) with Poisson distribution (or negative binomial if overdispersion occurred), a log link, robust (sandwich) standard errors, and a random intercept for country to account for unobserved between-country heterogeneity and to allow generalization beyond the 15 observed countries. Candidate covariates with p < 0.25 in univariable screening entered the multivariable GLMM. Model choice and adequacy were guided by the distribution of the outcome, residual diagnostics, the distribution of random effects, and Akaike’s Information Criterion. Two-sided tests were used with α = 0.05, and results are reported as incidence rate ratios (IRRs) with 95% confidence intervals (CIs).

Data were analyzed using StataNow 19.5SE (StataCorp., College Station, TX, United States), R ver. 4.4.1 (R Foundation for Statistical Computing, Vienna, Austria), and OriginPro 2025b (OriginLab, Northampton, MA, United States).

## 3 Results

### 3.1 Reimbursement requirements and procedures for FDCs used in hypertension

Reimbursement requirements for FDC medicines used in the treatment of hypertension varied across CEE countries, as detailed in Table 1. Most countries, except Croatia, Estonia, North Macedonia, and Serbia, have specific reimbursement regulations in place. In general, for an FDC medicinal product to be reimbursed, the pharmaceutical company has to submit a formal application. However, this requirement does not apply to Croatia. In Romania, FDCs may be automatically reimbursed if they consist solely of generic versions of innovative medicines that are already reimbursed; in such cases, no application is required. However, if this criterion is not fulfilled, submission of a formal reimbursement application becomes mandatory.

**TABLE 1:** Reimbursement requirements and procedures for FDCs used in hypertension treatment.

Reimbursement requirement or procedure	Albania	Bulgaria	Croatia	Czech Republic	Estonia	Hungary	Latvia	Lithuania	North Macedonia	Republic of Moldova	Montenegro	Poland	Romania	Serbia	Slovakia
Legal requirements for reimbursement of FDCs	Yes	Yes	No	Yes	No	Yes	Yes	Yes	No	Yes	Yes	Yes	Yes	No	Yes
Reimbursement procedures for FDCs differ from those for single- component medicines	No	Yes	No	Yes	No	Yes	Yes	Yes	No	Yes	No	Yes	Yes	Yes	No
FDCs follow the same reimbursement procedure as their closest equivalent, such as generic medicines	Yes	No	Yes	Yes	Yes	No	No	Yes	Yes	Yes	Yes	Yes	No	Yes	Yes
The FDC is subject to the same process as any new medicine without reimbursed equivalents (i.e., medicines containing the same active ingredient)	Yes	Yes	Yes	Yes	Yes	No	Yes	Yes	Yes	Yes	Yes	Yes	Yes	Yes	Yes
Formal reimbursement application required for refund by a third-party payer*	Yes	Yes	No	Yes	Yes	Yes	Yes	Yes	Yes	Yes	Yes	Yes	Yes/No^a^	Yes	Yes
Treatment adherence and persistence considered in the reimbursement process*	N/A	Yes	Yes	No	Yes	No	No	No	Yes	Yes	Yes	Yes	No	No	No
Clinical experts involved in reimbursement decision making*	Yes	Yes	Yes	No	Yes	No	Yes	No	Yes	Yes	Yes	Yes	No	Yes	Yes
Use of international guidelines on hypertension management in reimbursement decision making	Yes	Yes	Yes	Yes	Yes	Yes	Yes	Yes	Yes	Yes	Yes	Yes	Yes	Yes	Yes

* Applies to FDCs used in hypertension.

^a^ The FDC is automatically reimbursed if it contains only generics of innovative medicines that are already reimbursed.

Nine of the analyzed countries applied distinct procedures for FDCs compared to those used for single-component medicines. In contrast, six countries (i.e. Albania, Croatia, Estonia, North Macedonia, Montenegro, and Slovakia), treated FDCs in the same manner as any other medicinal product containing a single active pharmaceutical ingredient. In these cases, FDCs followed the same reimbursement pathway as the closest generic equivalent.

When a new FDC contains active substances not included in any already reimbursed medicinal product, it must undergo the full application process required for a new medication without reimbursed equivalents. This requirement was observed in Albania, Bulgaria, Croatia, Estonia, the Czech Republic, Latvia, Lithuania, North Macedonia, the Republic of Moldova, Montenegro, Poland, Romania, Serbia, and Slovakia.

Adherence and persistence considerations related to FDCs used in hypertension were included in reimbursement decision- making in seven of the analyzed countries, whereas the remaining seven countries did not incorporate these factors (no data for Albania). The questionnaire captured whether adherence and persistence were considered, but did not distinguish whether the supporting evidence was specific to the FDC under assessment or derived from broader evidence on analogous fixed-dose or free-equivalent combinations. Clinical experts were not involved in the reimbursement process in four countries: the Czech Republic, Hungary, Lithuania, and Romania. Notably, these same countries also did not consider adherence and persistence in their decision-making processes. In contrast, all countries reported taking international hypertension guidelines into account during reimbursement evaluations.

### 3.2 Pricing of reimbursed FDCs used in hypertension

Detailed data on pricing regulations for reimbursed FDCs used in hypertension across CEE countries are presented in Table 2. Several aspects were examined, including legal regulations on pricing, the use and conditions of price negotiations, the application of external reference pricing, variations in list price calculations, and the use of fixed or maximum prices for reimbursed FDCs.

**TABLE 2:** Pricing of reimbursed FDCs used in hypertension treatment.

Pricing requirement or procedure	Albania	Bulgaria	Croatia	Czech Republic	Estonia	Hungary	Latvia	Lithuania	North Macedonia	Republic of Moldova	Montenegro	Poland	Romania	Serbia	Slovakia
Legal regulations on pricing in the reimbursement process for FDCs	Yes	Yes	Yes	Yes	Yes	Yes	Yes	Yes	No	Yes	Yes	Yes	Yes	Yes	Yes
Price negotiation as part of the reimbursement process*	No	Yes	Yes	Yes	Yes	Yes	Yes	Yes	Yes	Yes	Yes	Yes	No	Yes	Yes
Requirement for price negotiation depends on the proposed price*	No	Yes	No	Yes	Yes	Yes	No	Yes	No	Yes	No	No	No	No	Yes
Requirement for price negotiations depends on the reimbursement status of FDC components*	No	No	No	No	No	No	Yes	No	No	No	No	No	No	No	No
Use of external reference pricing for FDCs*	Yes	Yes	Yes	Yes	Yes	No	No	Yes	N/A	No	No	No	Yes	No	Yes
Differences in the calculation of list prices for FDCs* vs. single components	No	No	N/A, no	No	No	No	No	Yes	No	No	No	Yes/No^a^	No	No	No
Fixed prices for reimbursed FDCs (official price equals list price)	Yes	No	Yes	No	Yes	Yes	Yes	No	N/A	No	Yes	Yes	No	Yes	No
Maximum prices for reimbursed FDCs (price does not exceed list price)	No	Yes	No	Yes	No	No	No	Yes	N/A	Yes	No	No	Yes	No	Yes

* Applies to FDCs used in hypertension.

^a^ A company can bypass the HTA process if it agrees to set the price at the level of the more expensive component; otherwise, this is not permitted.

Most countries have established regulations on pricing within their reimbursement systems, with the exception of North Macedonia. Similarly, price negotiation is a standard component of the reimbursement process in most countries, except for Albania and Romania. In Bulgaria, the Czech Republic, Estonia, Hungary, Lithuania, the Republic of Moldova, and Slovakia, the application of price negotiations depends on the proposed price of the product. In contrast, in Latvia, this depends on the reimbursement status of the FDC’s individual components. No such dependencies were identified in the remaining countries.

External reference pricing for FDCs used in hypertension was implemented in Albania, Bulgaria, Croatia, the Czech Republic, Estonia, Lithuania, Romania, and Slovakia. Among these, Lithuania was the only country that reported differences in the calculation of list prices for FDCs compared to single-component medicines. In Poland, the method for calculating list prices of FDCs depends on the pricing strategy adopted by the pharmaceutical company. If the company opts for the full HTA process, no difference in list price calculation is observed. However, if the company agrees to set the price at the level of the more expensive component, a different pricing pathway is applied. Fixed prices for reimbursed FDCs were reported in Albania, Croatia, Estonia, Hungary, Latvia, Montenegro, Poland, and Serbia. In contrast, maximum prices were used in Bulgaria, the Czech Republic, Lithuania, the Republic of Moldova, Romania, and Slovakia.

Overall, the results indicate substantial variation in pricing and reimbursement strategies for FDCs used in hypertension across different CEE countries, reflecting diverse regulatory frameworks and negotiation practices. These differences underscore the complexity of the pharmaceutical landscape in the region and highlight the need to adapt pricing and reimbursement policies to the specific regulatory and economic contexts of individual countries. While some similarities exist in the approaches adopted across CEE countries, the findings emphasize the importance of continued research and policy development to address these disparities and enhance the accessibility and affordability of hypertension treatments.

### 3.3 Health technology assessment of FDCs used in hypertension

Differences among CEE countries in the use, role, and content of HTA in the reimbursement process for FDCs used in hypertension are summarized in Supplementary Table S1. Only two countries (i.e., Albania and Croatia) do not require full HTA when applying for reimbursement of FDCs. In Croatia, only a budget impact analysis is required, while other HTA components are not utilized. Six countries—namely, the Czech Republic, North Macedonia, the Republic of Moldova, Montenegro, Romania, and Serbia—require certain HTA components when FDCs contain active substances that have already been reimbursed. However, the specific components required vary between countries. In the remaining countries, HTA requirements largely depend on the reimbursement status of the active substances included in the FDC. In Lithuania, although the standard HTA process is not mandatory, it may still be used. The process can be bypassed if the proposed price is at or below that of existing reimbursed equivalents.

An important component of the HTA process is the selection of an appropriate comparator, which serves as a benchmark for evaluating FDCs used in hypertension treatment. The choice of comparator can significantly influence both the HTA outcome and the reimbursement decision. The most commonly used comparators were the individual components of the FDC administered as separate medicinal products. This practice was reported in 13 countries. In contrast, reimbursed FDCs containing active substances from the same pharmacological classes were used less frequently, being reported in nine countries. The use of three or more comparator types was permitted in five countries, with two comparator types being most common: 1) the separate administration of the FDC’s active components, and 2) combinations of separate products from the same drug classes as those in the new FDC. Detailed results are presented in Supplementary Table S2.

The type of economic analysis applied in the HTA process depends on the approach used. If the effectiveness of an FDC is assumed to be equivalent to that of its individual active components, a full HTA may be omitted or at least simplified. In such cases, the active components—when administered as separate medicinal products—must have already undergone a reimbursement assessment. This practice was observed in most countries where HTA is a standard part of the reimbursement application process. Exceptions include Albania, Croatia, and North Macedonia, where an FDC used for hypertension is treated as any new medicinal product. In Albania and Croatia, HTA is not incorporated into the reimbursement process. In North Macedonia, no FDCs used in the treatment of hypertension are currently reimbursed.

In Poland, specific reimbursement regulations allow the marketing authorization holder to choose between two pricing pathways for FDCs. A simplified option is available if the holder agrees to set the FDC price at the level of the more expensive previously reimbursed component. Alternatively, the company may pursue a full HTA, which allows for the possibility of setting a higher price. However, this approach requires strong supporting evidence from a cost-utility analysis.

Only seven countries required clinical analyses based on systematic reviews as part of the reimbursement process for FDCs. This is particularly noteworthy, as systematic reviews assess health outcomes of the evaluated technology and provide high-quality evidence on its effectiveness and safety in a specific population compared to appropriate comparators. In the Czech Republic and Latvia, clinical analyses are required when not all components of the FDC are already reimbursed. In Poland, such analysis is mandatory if the pharmaceutical company seeks a higher price than that of the more expensive previously reimbursed component. In the Republic of Moldova, clinical analysis is required when the FDC or the specific strength intended for reimbursement is not yet listed on the national positive list.

### 3.4 Availability of FDCs used in hypertension

A comprehensive overview of the availability of FDCs used in hypertension treatment across various CEE countries is presented in Supplementary Table S3. The data are organized into several key domains, including the time from application submission to inclusion on the reimbursement list; the existence of limit groups with shared reimbursement thresholds for similar medicines; whether reimbursement limits are set individually for each medicine; the option for patients to purchase FDCs at a higher price if it exceeds the reimbursement limit; the absence of reimbursement when prices surpass the limit; differences in patient co-payments between FDCs and single-component medicines; and the frequency of updates to the list of reimbursed medicines.

The time from application submission to inclusion on the reimbursement list varied significantly across CEE countries, ranging from less than 3 months in Latvia and Lithuania to more than 12 months in Albania and Romania. In most countries, the process typically took between 6 and 8 months. Another important factor is the use of limit groups, which assign a common reimbursement threshold to similar types of medicines. These groups are used to control public spending on medicines by setting a maximum reimbursable price for all drugs within the group. Such groups were reported to influence the reimbursement of FDCs used in hypertension treatment in Croatia, Estonia, Hungary, the Republic of Moldova, Montenegro, Poland, Romania, and Serbia. In contrast, no limit groups were reported in Albania, Bulgaria, the Czech Republic, Latvia, Lithuania, North Macedonia, and Slovakia.

Reimbursement limits—defined as the maximum amount reimbursed by the public payer for a given medicine—were set individually for each drug in only a few countries, namely, the Republic of Moldova, Montenegro, and Serbia. In most countries, patients are allowed to purchase FDCs at a higher price than the reimbursement limit by paying the difference between the retail price and the reimbursed amount. An exception is Bulgaria, where no reimbursement limit is applied. The absence of reimbursement when the price exceeds the limit was reported only in Hungary and Lithuania. In Lithuania, reimbursement limits also serve as a mechanism for controlling patient out-of-pocket costs by setting a maximum annual allowable co-payment.

Patient co-payment for FDCs used in hypertension treatment differs from that for single-component medicines in Bulgaria, the Czech Republic, Lithuania, the Republic of Moldova, and Serbia. In the remaining countries, a uniform co-payment structure is maintained regardless of whether the medicine is a combination or a single-component product. In the Republic of Moldova, the patient’s co-payment for reimbursed FDCs does not necessarily equal the combined co-payments of the individual components when reimbursed separately. Consequently, the final retail price and the patient’s out-of-pocket payment may vary depending on the price level accepted for the FDC.

In Slovakia, the process of determining reimbursement for FDCs can be complex. When reference groups include medicines with the same amounts of active ingredients as the FDC, the reimbursement amount is calculated as the sum of the maximum reimbursable amounts for each component. However, if the reference groups contain different amounts of active ingredients, the reimbursement is determined by converting those amounts to the closest equivalent dose. In cases of uncertainty, the nearest lower equivalent is selected. Nonetheless, this procedure has certain limitations. If the lowest maximum retail price of a medicine in a public pharmacy—when converted to the standard dose of the active ingredient—is lower than the reimbursement amount calculated using these methods, the lower price is applied.

The frequency of updates to the reimbursement list varied considerably across CEE countries. Very frequent updates, occurring monthly or every 2 months, were reported in Bulgaria, Latvia, and Slovakia. In contrast, extremely rare updates (less than once a year) were observed in Albania and the Czech Republic. A moderate frequency of changes—approximately every three or four months—was reported in Estonia, the Republic of Moldova, Poland, and Serbia. In Hungary, Lithuania, North Macedonia, Montenegro, and Romania, updates typically occurred once or twice per year. In Hungary, the frequency of updates is linked to the time elapsed since a medicine’s patent expiration. During the first year after patent expiry, reimbursement updates occur quarterly, and from the second year onward, they occur biannually. In Croatia, reimbursement list updates are not frequent. The prices of all medicines, including FDCs, are reviewed annually and adjusted if necessary. Although the Medicines Committee of the Croatian Health Insurance Fund (*Hrvatski zavod za zdravstveno osiguranje* [HZZO]) convenes once a month, no changes are made unless a new FDC is added to the reimbursement list. In such cases, price adjustments may occur.

Overall, the data presented in Table 1 as well as Supplementary Table S1 highlight the diverse regulatory and reimbursement landscapes for hypertension treatments across CEE countries, reflecting substantial differences in both accessibility and the financial burden placed on patients.

Various factors that influence the level of patient co-payment for FDCs used in the treatment of hypertension across the 15 CEE countries are presented in Supplementary Table S4. These factors include therapeutic indication, patient income, duration of medicine use, medicine price, total public expenditure on reimbursed medicines, the patient’s insurance plan, and the type and number of active substances in the medicine.

Therapeutic indication was one of the two most commonly considered factors in determining patient co-payment levels, with all countries except the Czech Republic, Montenegro, and Romania taking it into account. In contrast, patient income was considered less frequently, included in the co-payment assessment only in Albania, Lithuania, and Romania. Another infrequently considered factor was the duration of medicine use, which was taken into account only in Albania, North Macedonia, and Poland.

The price of the medicine influenced co-payment levels in all analyzed countries except Estonia and Hungary, making it the most common determining factor. Total expenditure on reimbursed medicines was taken into account in Albania and Lithuania, reflecting a broader approach to managing healthcare costs.

The type and number of active substances in a medicine generally do not influence the level of patient co-payment for FDCs used in hypertension across CEE countries, with the exception of Poland. These findings highlight the different approaches adopted by the analyzed countries in determining patient co-payment levels for FDCs used in hypertension, reflecting variations in healthcare policies and economic considerations. The variability of these factors underscores the complexity of healthcare systems and the need for tailored strategies to ensure equitable access to essential medicines for hypertension management. For example, in the Republic of Moldova, in addition to therapeutic indication and medicine price, several other factors are considered, including the Ministry of Health’s prioritization of severe diseases, target patient groups, alignment with national health strategies, the intended patient population (e.g., children, pregnant women), and whether the medicine is included in the National Essential Medicines List. In Poland, an additional factor influencing co-payment determination is the inclusion of special patient groups, such as individuals aged 75 years or older, pregnant women, or minors.

Overall, Supplementary Table S4 provides a comprehensive overview of the multifaceted factors influencing patient co-payment levels for FDCs used in hypertension. It highlights therapeutic indication and medicine price as the primary determinants. The limited consideration of patient income and duration of medicine use indicates potential areas for policy enhancement, aiming to improve affordability and access to hypertension treatments.

A detailed summary of the availability and reimbursement status of FDCs containing two active components for hypertension treatment across 15 CEE countries is presented in Table 3. The most frequently reimbursed categories of FDCs used in hypertension across the analyzed countries were those combining ARBs with CCBs, ACEIs with diuretics, ARBs with diuretics, and ACEIs with CCBs. These combination categories are reimbursed in almost all analyzed countries except North Macedonia, where these FDCs are available but not reimbursed—as no FDCs used in hypertension are reimbursed there. Additionally, ARBs with CCBs and ACEIs with CCBs are not reimbursed in the Republic of Moldova. In contrast, FDCs combining CCBs with diuretics, ACEIs with beta-blockers, CCBs with beta-blockers, and diuretics with beta-blockers are less commonly reimbursed. For example, FDCs combining CCBs with diuretics are reimbursed in Bulgaria, Croatia, Hungary, Latvia, Lithuania, and Serbia, but not in Albania, the Czech Republic, Estonia, North Macedonia, the Republic of Moldova, Montenegro, Poland, Romania, or Slovakia.

**TABLE 3:** Two-component reimbursed FDCs used in hypertension treatment.

Classes of medications	Albania	Bulgaria	Croatia	Czech Republic	Estonia	Hungary	Latvia	Lithuania	North Macedonia*	Republic of Moldova	Montenegro	Poland	Romania	Serbia	Slovakia
ARB + CCB	+	+	+	+	+	+	+	+	Available not reimbursed	-	+	+	+	+	+
ACEI + DIU	+	+	+	+	+	+	+	+	Available not reimbursed	+	+	+	+	+	+
ARB + DIU	+	+	+	+	+	+	+	+	Available not reimbursed	+	+	+	+	+	+
ARB + ACEI	-	-	-	-	-	-	-	-	-	-	-	-	-	-	-
ACEI + CCB	+	+	+	+	+	+	+	+	Available not reimbursed	-	+	+	+	+	+
CCB + DIU	-	+	+	-	-	+	+	+	-	-	-	-	-	+	-
ARB + BB	-	-	-	-	-	-	-	-	-	-	-	-	-	-	-
ACEI + BB	-	+	+	+	-	+	+	+	-	-	-	-	-	-	+
CCB + BB	-	+	+	+	-	Available not reimbursed	+	+	-	-	-	-	-	-	+
DIU + BB	+	+	+	+	-	Available not reimbursed	+	+	-	-	-	-	+	+	+

* No FDCs used in hypertension treatment are reimbursed in North Macedonia; some FDCs are available but not reimbursed. Symbols: +, reimbursed; –, not reimbursed.

Abbreviations: ACEI, angiotensin-converting enzyme inhibitor; ARB, angiotensin receptor blocker; BB, beta-blocker; CCB, calcium channel blocker; DIU, thiazide and thiazide-like diuretic.

Other less frequently reimbursed categories of FDCs included those combining ACEIs with beta-blockers and CCBs with beta-blockers. Specifically, the combinations of ACEIs and beta-blockers are reimbursed in Bulgaria, Croatia, the Czech Republic, Hungary, Latvia, Lithuania, and Slovakia, but not in Albania, Estonia, North Macedonia, the Republic of Moldova, Montenegro, Poland, Romania, or Serbia. Similarly, FDCs combining CCBs with beta-blockers are reimbursed in Bulgaria, Croatia, the Czech Republic, Latvia, Lithuania, and Slovakia, but not in Albania, Estonia, Hungary, North Macedonia, the Republic of Moldova, Montenegro, Poland, Romania, or Serbia.

The data also highlight considerable variability in the reimbursement of FDCs of diuretics with beta-blockers. These combinations are reimbursed in Albania, Bulgaria, Croatia, the Czech Republic, Latvia, Lithuania, Romania, Serbia, and Slovakia, but not in Estonia, Hungary, North Macedonia, the Republic of Moldova, Montenegro, or Poland. This variability reflects, at least to some extent, differences in national health policies and economic considerations. For example, although Albania, Croatia, and Slovakia apply the same reimbursement procedures for FDCs as for single-component medicines, these combinations are nonetheless reimbursed—similarly to Bulgaria, the Czech Republic, Latvia, Lithuania, Romania, and Serbia, where a different reimbursement approach is employed.

The comparison presented in Table 3 was performed at the therapeutic class-combination level and was not adjusted for the number of individual substance-level combinations registered or marketed within each category in each country. Therefore, the lower reimbursement frequency observed for some categories may partly reflect fewer available substance-level combinations rather than reimbursement restrictiveness alone. Overall, the data underscore the widespread availability and reimbursement of selected FDC categories, particularly those combining ARBs with CCBs, ACEIs with diuretics, ARBs with diuretics, and ACEIs with CCBs, across the CEE region. In contrast, reimbursement was more limited for categories such as CCBs with diuretics, ACEIs with beta-blockers, CCBs with beta-blockers, and diuretics with beta-blockers. Notably, ARB + ACEI and ARB + beta-blocker combinations were not reimbursed in any of the countries included in the analysis.

### 3.5 The total number of reimbursed FDCs used in hypertension by country

Total number of reimbursed FDCs used in hypertension ranged from 0 to 78 different active ingredient combinations and doses. The total numbers in each country were as follows: North Macedonia (0), Republic of Moldova (4), Albania (24), Montenegro (33), Romania (35), Serbia (50), Estonia (52), Czech Rep. (61), Poland (62), Croatia (66), Lithuania (66), Bulgaria (68), Hungary (68), Slovakia (71), Latvia (78). The total number of reimbursed FDC classes did not differ significantly between countries (p = 0.407). However, there was a significant difference between countries in the total number of separate FDCs reimbursed (p < 0.001). The variability in reimbursement status suggests that while some countries have adopted a more inclusive approach to the reimbursement of FDCs for hypertension, others maintain more restrictive policies. These differences may be driven by factors such as national healthcare budgets, the prioritization of hypertension treatment, and the perceived cost-effectiveness of various FDCs.

The number of FDCs reimbursed in a country was monotonically associated with: the requirements of HTA in the reimbursement process of FDCs containing previously reimbursed components (moderate correlation with ρ = 0.641, p = 0.011; Supplementary Figure S1), and the time from application to the inclusion in the reimbursement list (fair correlation with ρ = −0.548, p = 0.041; Supplementary Figure S2).

Regression analyses confirmed some of the results of Spearman correlation analysis. The time from application to reimbursement was not found to be statistically significant in univariate GLMM (p = 0.553). However, GLMM suggested that countries with less strict requirements for FDCs containing already reimbursed components tend to have a higher number of reimbursed FDCs. The countries that do not require HTA or make the HTA requirement contingent on the manufacturer’s proposed price (p = 0.001) and countries that do not demand additional comparators within HTA (e.g., polytherapy with agents other than the FDC components) have a higher number of reimbursed FDCs (p = 0.010). Multivariable GLMM suggested that the consideration of treatment adherence/compliance and persistence in the reimbursement process was not significantly associated with the number of reimbursed FDCs (p = 0.085); likewise, setting a reimbursement limit for FDCs individually, independently from the limits of its components was not statistically significant (p = 0.447; Table 4).

**TABLE 4:** Determinants of the number of FDCs reimbursed: Univariable and multivariable generalized linear mixed model.

Variable	​	Univariable model		Multivariable model	
	*Level/overall test*	*IRR (95% CI)*	*p value*	*IRR (95% CI)*	*p value*
Treatment adherence and persistence considered in the reimbursement process	No	*reference*	-	*reference*	-
	Yes	0.47 (0.18–1.23)	0.124	0.59 (0.32–1.08)	0.085
HTA components required for the reimbursement of FDCs containing previously reimbursed components	CMA	*reference*	-	*reference*	-
	Full HTA	0.38 (0.12–1.26)	0.115	0.97 (0.31–3.02)	0.955
	Full HTA if price > sum of component prices	0.10 (0.02–0.51)	0.006	0.44 (0.08–2.36)	0.336
	None	1.37 (0.59–3.22)	0.467	5.95 (1.76–20.16)	0.004
	None if price < sum of component prices	1.83 (0.71–4.73)	0.212	2.03 (0.77–5.30)	0.150
	Simple BIA if price < the most expensive component; full HTA otherwise	1.61 (0.42–6.14)	0.487	3.03 (0.81–11.28)	0.099
	*overall test*	-	0.003	-	0.001
Other combinations of medicines in separate products (from the same drug classes as the new FDC) used as comparator	N/A	*reference*	-	*reference*	-
	No	1.40 (0.39–5.04)	0.609	3.82 (1.44–10.12)	0.007
	Yes	0.36 (0.08–1.57)	0.173	1.08 (0.48–2.43)	0.846
	*overall test*	-	0.042	-	0.010
Reimbursement limit set individually for FDCs, independently of its components	No	*reference*	-	*reference*	-
	Yes	0.46 (0.14–1.55)	0.209	1.51 (0.52–4.40)	0.447

Abbreviations: FDCs, fixed-dose combinations; IRR, incidence rate ratio; N/A, not available (e.g., no HTA required); CMA, cost-minimization analysis; BIA, target population size calculation and/or budget-impact analysis; HTA, health technology assessment; CI, confidence interval.

Reference, baseline level for each categorical predictor (IRRs for other levels are relative to this level); Overall test, Wald χ^2^ test for the predictor as a whole (i.e., that all non-reference coefficients equal zero).

### 3.6 Public spending on reimbursement across CEE countries

The data presented in Supplementary Table S5 provide a comparative overview of public spending on reimbursed medicines across the CEE countries, expressed in purchasing power standard (PPS). The table covers the years 2021, 2022, and 2023, and includes information on total public spending on reimbursed medicines, as well as specific expenditures related to hypertension treatments and FDCs used in hypertension therapy. While the table includes all the analyzed countries, data availability varies by year and by country, making it difficult to conduct a fully comprehensive comparison.

In 2021, Poland recorded the highest total public spending on reimbursed medicines, exceeding 3.1 billion PPS units, followed by Slovakia (approximately 2.08 billion) and Croatia (just over 2 billion). In contrast, countries such as North Macedonia, Albania, Estonia, and Montenegro reported substantially lower expenditures, with North Macedonia spending just over 101 million PPS units and Montenegro slightly above 210 million PPS units. Notably, data for the Republic of Moldova and Romania were unavailable for this year. When focusing on hypertension-related reimbursements, Hungary reported the highest spending at nearly 165 million PPS units, followed by Croatia and Slovakia. Expenditure on FDCs for hypertension was also highest in Hungary (65 million PPS units), Croatia (59 million PPS units), and Slovakia (33 million PPS units), indicating a strong policy focus on combination therapies in these countries. In the case of Serbia, available data encompassed both reimbursed and out-of-pocket spending, as separate figures were not reported. Total spending there amounted to approximately 2.25 billion PPS units, with 241 million PPS units allocated to hypertension-related medicines and 71 million PPS units to FDCs for hypertension.

In 2022, most CEE countries experienced an increase in total public spending on reimbursed medicines. For instance, Bulgaria’s expenditure rose from approximately 919 million PPS units in 2021 to nearly 973 million PPS units in 2022, while the Czech Republic increased its spending from around 640 million to over 721 million PPS units. In contrast, Poland recorded a slight decline, with total expenditure decreasing from 3.12 billion to 2.96 billion PPS units. Spending on hypertension-related medicines generally declined or remained stable across most countries. Hungary reported nearly 158 million PPS units. Serbia reported approximately 263 million PPS units, but this figure combined reimbursed and out-of-pocket spending and is therefore not directly comparable. In comparison, Croatia and the Czech Republic registered modest decreases. The spending patterns for FDCs largely mirrored those observed for hypertension medicines, with Estonia and Slovakia noting slight increases in reimbursement spending. Hungary maintained consistently high investment levels in FDC therapies throughout 2022.

By 2023, data availability became more limited, yet several notable trends persisted. Bulgaria’s total public spending on reimbursed medicines rose significantly, exceeding 1.1 billion PPS units, while Hungary’s expenditure continued to increase, reaching nearly 1.95 billion PPS units. The Czech Republic also experienced a substantial rise, with spending approaching 892 million PPS units, and Poland’s expenditure surged to over 3.5 billion PPS units—the highest level recorded in the dataset. However, data for Albania, Croatia, Romania, and Serbia were not available for this year. In terms of hypertension-related reimbursements, Hungary remained the leading spender, allocating approximately 150 million PPS units, followed by Slovakia and the Czech Republic. Expenditure on FDCs for hypertension remained notably high in Hungary (over 62 million PPS units) and Slovakia (over 36 million PPS units).

Share of FDCs in hypertension-related reimbursement spending also varies across countries. It is noteworthy that in several CEE countries, FDCs account for approximately half of total reimbursement spending on hypertension medicines. For instance, Albania allocated 48% of its hypertension reimbursement expenditures to FDCs in 2022 and 52% in 2023. Bulgaria reported similar figures, with 48% in 2021 and 49% in both 2022 and 2023. Croatia spent nearly 48% on FDCs in 2022, while Estonia allocated 55% in 2021 and 68% in 2022. Latvia maintained consistently high levels, reporting 55% in 2021–2022 and 56% in 2023. Lithuania allocated approximately 49% in 2022 and 50% in 2023. Across the remaining countries with available data, the share of FDCs in hypertension-related reimbursement spending ranged from 23% in the Czech Republic (2021–2023) to 68% in Estonia (2022).

## 4 Discussion

This study examined reimbursement policies for fixed-dose combinations (FDCs) used in hypertension across Central and Eastern European (CEE) countries. The findings reveal substantial differences in how these countries regulate, evaluate, and reimburse FDCs, despite broadly similar clinical recommendations and increasing global emphasis on single-pill combination therapy (McEvoy et al., 2024). This diversity mirrors well-documented variations in European reimbursement systems for other complex therapeutic areas, including orphan drugs, where national-level policies and institutional practices often lead to heterogeneous access to medicines (Kawalec et al., 2016; Malinowski et al., 2018). Our analysis adds to this body of evidence by demonstrating that comparable disparities are present within the hypertension domain, particularly for FDCs—medicines intended to simplify treatment regimens and improve adherence.

Across the included countries, the policy requirements surrounding FDC reimbursement ranged from minimal procedural demands to extensive, multi-component HTA. Such divergence represents two extremes of regulatory design: some systems adopt flexible pathways that treat FDCs similarly to their single-component predecessors, while others require full reassessment despite shared active ingredients. Prior research has shown that policy stringency influences access outcomes, especially where reimbursement decisions rely heavily on detailed economic or clinical evidence (Nicod et al., 2019). Our findings align with this literature and indicate that the regulatory burden placed on manufacturers is not uniform across countries, which may lead to unequal uptake of combination therapies.

Our observation reinforces previous arguments that procedural design can either facilitate or restrict patient access to medicines, depending on how evidence demands are calibrated (Blonda et al., 2021; Blonda et al., 2022). In many cases, stricter HTA processes may be justified for novel or high-cost innovations; however, in the context of FDCs whose components already exist within the reimbursement system, excessively rigid requirements may not reflect the incremental evidence available. Two regulatory aspects—HTA requirements for products containing already reimbursed components and comparator expectations—appear particularly important in shaping the number of reimbursed FDCs. Simplifying these procedures, while maintaining appropriate standards of evaluation, could potentially reduce disparities and improve alignment with clinical guidelines.

The use of GLMMs has important limitations. With only 15 countries, the variances of both fixed effect and the random intercept were estimated with considerable uncertainty. The random effect primarily served as a device to accommodate excess between-country variability, while also substantially improving the fit to the observed data. We re-estimated the models using models without random effects (not shown); these yielded qualitatively similar results, suggesting that our main conclusions are not driven by the specific choice of modelling framework. Another important limitation is that the number of registered and marketed antihypertensive substance-level combinations available in each country but not reimbursed was not included as a potential confounder. Consequently, cross-country differences in market availability may have influenced the observed number of reimbursed FDCs, and the associations between reimbursement-policy features and FDC availability should be interpreted with caution.

We found no evidence of missing-data-related sparsity or clinically problematic outcome sparsity in our models. Nevertheless, because each country contributed only one observation, and some categorical levels and covariate patterns were represented by very few countries, the model should be interpreted as an exploratory association model rather than a confirmatory multivariable model. The random intercept should not be interpreted as estimating within-country correlation; instead, in this setting it functions as an exploratory adjustment for residual between-country heterogeneity and/or overdispersion.

National reimbursement systems are complex, and country-specific legal or institutional exceptions could not be fully represented in the statistical models. The categorical variables used in the analysis were simplified indicators of broad reimbursement-policy features, selected because they were consistently identifiable across countries. These variables should not be interpreted as complete descriptions of national legal frameworks. Because only 15 countries were included, more granular legal coding would have increased sparsity and overfitting; therefore, the results should be interpreted as exploratory associations between broad reimbursement-policy characteristics and FDC availability, rather than as evidence of direct causal effects of specific legal provisions.

An important aspect emerging from our analysis is the role of comparator choice in HTA evaluations. In several countries, the most common comparator for an FDC was the separate administration of its individual components, while others also considered broader therapeutic alternatives. Prior studies have highlighted the significant impact of comparator selection on reimbursement outcomes across Europe, as inappropriate benchmarks can disadvantage therapies by inflating perceived budget impact or distorting cost-effectiveness ratios (Allen et al., 2017).

Countries vary in their use of fixed prices, maximum allowable prices, external reference pricing, and price negotiations. These mechanisms influence reimbursement outcomes not only through direct cost calculations but also through manufacturers’ willingness to submit applications. Earlier research in Europe has shown that economic restrictions, differences in public budgets, and negotiation processes contribute to inequities in access to medicines, even when clinical evidence is aligned (Picavet et al., 2012).

Importantly, some countries do not incorporate adherence or persistence into reimbursement criteria, nor do they consistently involve clinical experts in decision-making. This stands in contrast to growing evidence demonstrating that FDCs improve adherence compared with free combinations, thereby enhancing long-term treatment success (Du et al., 2018; Schmieder et al., 2023). A meta-analysis published in 2023 demonstrated that the likelihood of adherence with FDCs was approximately 29% greater than that of free associations (RR ∼1.29) (Wei et al., 2023). Earlier work has shown that many reimbursement frameworks do not sufficiently capture patient-centered value dimensions such as convenience, reduced pill burden, or improved quality of life (Dupont and Van Wilder, 2011; Zimmermann et al., 2021). Our findings suggest that these gaps persist in CEE decision-making processes and that this could potentially hinder the adoption of therapeutically valuable FDCs. Given the chronic nature of hypertension and the demonstrated benefits of FDC therapies for adherence, aligning reimbursement processes with evidence-based clinical practice should be a priority.

In Europe, collaborative initiatives such as the BeNeLuxA partnership have demonstrated that voluntary cooperation across countries can streamline assessment processes, enhance transparency, and promote more consistent pricing outcomes (Vogler et al., 2021). While the present study focuses on CEE countries not currently participating in BeNeLuxA, the underlying model offers relevant lessons. Shared evaluations, joint HTA components, or common comparator frameworks could help reduce procedural duplication and ease the burden on smaller markets, consistent with the direction of the EU HTA framework (European Parliament and Council of the European Union, 2021).

Furthermore, this study echoes earlier conclusions that not all FDCs receive equal treatment within national reimbursement systems. As observed in previous research on therapies with high unmet needs, administrative and procedural differences can overshadow clinical priorities (Sarnola et al., 2018; Malinowski et al., 2019). Although FDCs are recommended by major hypertension guidelines, the alignment between clinical recommendations and national reimbursement practices, understood as a vast range of available combinations, remains inconsistent. This gap may lead to suboptimal treatment patterns and hinder efforts to improve hypertension control across the region.

## 5 Conclusion

Our study identified substantial differences in reimbursement, pricing, availability, and HTA processes for fixed-dose combinations used in hypertension treatment across Central and Eastern European countries. These differences may contribute to unequal access to single-pill combination therapies, despite contemporary clinical guidelines supporting combination treatment for many patients with hypertension.

The most important policy-relevant finding is that countries with less burdensome HTA requirements for FDCs composed of already known and reimbursed active substances tended to reimburse more such products. This suggests that reimbursement systems could better support patient access by applying proportionate assessment pathways to well-justified, guideline-supported combinations with a clear clinical rationale. Such streamlining may reduce unnecessary procedural duplication while maintaining evidence-based decision-making and better aligning reimbursement practice with clinical recommendations.

The emerging common EU HTA framework may further support consistency in clinical evidence assessment and reduce duplication across national HTA systems. However, its role should be understood as supportive, since final conclusions on clinical added value, pricing, and reimbursement remain within national competence. Future policy efforts should therefore focus on balancing rigorous evaluation with proportionate evidence requirements for established, clinically justified, and guideline-aligned FDCs.

## Data Availability

All data supporting the results of this study are included in the article and its Supplementary material. Additional information is available from the corresponding author upon reasonable request.
