# Supplementary material for: Availability and reimbursement of fixed-dose combination medicines for hypertension treatment in Central and Eastern Europe
Source: Front Pharmacol. 2026 Oct 8;17:1889485. doi: 10.3389/fphar.2026.1889485 (PMC13687808; doi:10.3389/fphar.2026.1889485)
Supplement: Supplementary file 1 [file Table1.docx]

Supplementary Material

## 1.1 Supplementary Tables

Supplementary Table 1. Supporting data required in the reimbursement process of FDCs used in hypertension treatment.

|  | Albania | Bulgaria | Croatia | Czech Republic | Estonia | Hungary | Latvia | Lithuania | North Macedonia | Republic of Moldova | Montenegro | Poland | Romania | Serbia | Slovakia |
| --- | --- | --- | --- | --- | --- | --- | --- | --- | --- | --- | --- | --- | --- | --- | --- |
| HTA or its component(s) included in the reimbursement application | No | Yes | BIA | Yes | Yes | Yes | Yes | Yes | Yes | Yes | Yes | Yes | Yes | Yes | Yes |
| HTA components required for the reimbursement of FDCs containing previously reimbursed components | N/A | None | None | CA, SCC, BIA | None | None^1^ | None^1^ | None^1^ | FULL | CA, SCC, BIA^1^ | CMA | None/FULL^2^ | CMA | CMA | None |
| HTA components required for the reimbursement of FDCs containing new active substances | N/A | FULL | BIA | FULL | FULL | FULL | FULL | FULL | FULL | FULL | FULL | FULL | FULL | FULL | FULL |
| Type of economic analysis included in HTA dossier for new FDCs in hypertension with previously registered components | N/A | CMA | BIA | CMA, CUA, SCC, BIA | CMA, CEA, CUA, BIA | CUA | CMA, CEA | CMA | FULL | FULL | FULL | FULL | CMA | CMA, BIA | CMA, CEA, CUA, |
| Clinical analysis based on a systematic review required in the reimbursement process | No | Yes | No | No^3^ | Yes | No | No^3^ | No | Yes | Yes | Yes | No^4^ | N/A | Yes | Yes |

^1^ When the price of an FDC does not exceed the total price of the reimbursed components.

^2^ If the price is set on the level of the more expensive component BIA; FULL HTA if a pharmaceutical company seeks a higher price than that of the more expensive component.

^3^ If not all components are reimbursed, the answer is ‘yes’.

^4^ If a pharmaceutical company seeks a higher price than that of the more expensive component, the answer is ‘yes’.

Abbreviations: BIA, target population size calculation and/or budget-impact analysis; CA, clinical analysis; CEA, cost-effectiveness analysis; CMA, cost-minimization analysis; CUA, cost-utility analysis; FULL, full economic analysis; N/A, not available; SCC, simple cost comparison -a method that compares the costs of two interventions to identify the less expensive option.

Supplementary Table 2. Comparator treatments used in the reimbursement process for new FDCs for hypertension.

|  | Albania | Bulgaria | Croatia | Czech Republic | Estonia | Hungary | Latvia | Lithuania | North Macedonia | Republic of Moldova | Montenegro | Poland | Romania | Serbia | Slovakia |
| --- | --- | --- | --- | --- | --- | --- | --- | --- | --- | --- | --- | --- | --- | --- | --- |
| Components of the new FDC in separate products | N/A | Yes | N/A | Yes | Yes | Yes | Yes | Yes | Yes | Yes | Yes | Yes | Yes | Yes | Yes |
| Other reimbursed FDCs containing active substances from the same drug classes | N/A | Yes | N/A | No | Yes | No | No | No | Yes | Yes | Yes | Yes | Yes | Yes | Yes |
| Other reimbursed FDCs containing active substances (no limitation on drug class) | N/A | No | N/A | No | Yes | No | No | No | No | No | No | No | Yes | No | No |
| Other combinations of medicines in separate products (from the same drug classes as the new FDC) | N/A | No | N/A | No | Yes | No | No | No | Yes | Yes | No | No | No | No | Yes |
| Other combinations of medicines in separate products (no limitation on drug class) | N/A | No | N/A | No | Yes | No | No | No | No | No | No | No | Yes | No | No |

Abbreviations: N/A, not available

Supplementary Table 3. Availability aspects of FDCs used in hypertension treatment.

|  | Albania | Bulgaria | Croatia | Czech Republic | Estonia | Hungary | Latvia | Lithuania | North Macedonia | Republic of Moldova | Montenegro | Poland | Romania | Serbia | Slovakia |
| --- | --- | --- | --- | --- | --- | --- | --- | --- | --- | --- | --- | --- | --- | --- | --- |
| Time from application to inclusion in reimbursement list, months | 9-12> | 1-2-6^1^ | 3-5 | 6-8 | 6-8 | No info | <3 | <3 | 6-8 | 6-8 | 6-8 | 3-12 | 6-8 (12>)^2^ | 3-5 | 6-8 |
| Limit groups with a shared limit for similar reimbursed medicines affecting FDCs used in hypertension | No | No | Yes | No | Yes | Yes | No | No | No | Yes | Yes | Yes | Yes | Yes | No |
| Reimbursement limit set individually for each drug affecting FDCs used in hypertension | No | No | No | No | No | No | No | No | No | Yes | Yes | No | No | Yes | No |
| The patient can buy FDCs used for hypertension at a higher price than the limit but covers the difference between the price and the limit. | Yes | N/A | Yes | Yes | Yes | Yes | Yes | Yes^3^ | Yes | Yes | Yes | Yes | Yes | Yes | Yes |
| No reimbursement is provided if the price exceeds the limit: the patient covers 100% of the price. | No | N/A | No | No | No | Yes | No | Yes | No | No | No | No | No | No | No |
| Patient co-payment for FDCs used for hypertension is different from that for single-component medicines | No | Yes | No | Yes | No | No | No | Yes | No | Yes | No | No | No | Yes | No^4^ |
| Frequency of changes to the list of reimbursed medicines^5^ | Very rarely | Very frequent | Rarely | Very rarely | Frequently | Rarely^6^ | Very frequent | Rarely | Rarely | Frequently | Rarely | Frequently | Rarely | Frequently | Very frequent |
| Experts' subjective assessment of the availability level of FDCs* | High/ Moderate | Moderate | Moderate | High | High | Moderate | High | High | Low | Moderate | High | Moderate | Moderate | Moderate | High |

* Applies to FDCs used in hypertension.

^1^ For FDCs containing new active substances with an HTA evaluation, 180 days; for FDCs containing active substances with well-established use as single products and representing the first combination to apply for reimbursement – 60 days, for generic FDC products, 30 days.

^2^ If FDC is composed of single products that have not been previously reimbursed, time from application to inclusion can be higher than 12 months

^3^ If FDC is included in the price list.

^4^ See section 3.4 for a detailed explanation.

^5^ Very frequently (every month or every two months); frequently (every three or four months); rarely (once or twice per year); very rarely (less than once a year)

^6^ In the first year after patent expiry, the reimbursement is changed quarterly (4 times a year). From the second year, the reimbursement is changed twice a year.

Abbreviations: N/A, not available.

Supplementary Table 4. Factors influencing the level of patient co-payment for FDCs used in hypertension treatment.

|  | Albania | Bulgaria | Croatia | Czech Republic | Estonia | Hungary | Latvia | Lithuania | North Macedonia | Republic of Moldova | Montenegro | Poland | Romania | Serbia | Slovakia |
| --- | --- | --- | --- | --- | --- | --- | --- | --- | --- | --- | --- | --- | --- | --- | --- |
| Therapeutic indication | Yes | Yes | Yes | - | Yes | Yes | Yes | Yes | Yes | Yes | - | Yes | - | Yes | Yes |
| Patient income | Yes | - | - | - | - | - | - | Yes | - | - | - | - | Yes | - | - |
| Duration of medicine use | Yes | - | - | - | - | - | - | - | Yes | - | - | Yes | - | - | - |
| Price of the medicine | Yes | Yes | Yes | Yes | - | - | Yes | Yes | Yes | Yes | Yes | Yes | Yes | Yes | Yes |
| Total expenditure on reimbursed medicines | Yes | - | - | - | - | - | - | Yes | - | - | - | - | - | - | - |
| Insurance plan | - | - | - | - | - | - | - | - | - | - | - | - | - | - | - |
| Type and number of active substances in the medicine | - | - | - | - | - | - | - | - | - | - | - | Yes | - | - | - |
| Other | - | - | - | - | - | - | - | - | - | Yes^1^ | - | Yes^1^ | - | - | - |

^1^ See section 3.4 for a detailed explanation.

Supplementary Table 5. Spending on reimbursement Real Expenditure - Purchasing power standard (EU27_2020=1).

|  | Albania | Bulgaria | Croatia | Czech Republic | Estonia | Hungary | Latvia | Lithuania | North Macedonia | Republic of Moldova | Montenegro | Poland | Romania | Serbia* | Slovakia |
| --- | --- | --- | --- | --- | --- | --- | --- | --- | --- | --- | --- | --- | --- | --- | --- |
| 2021 | | | | | | | | | | | | | | | |
| Total public spending on reimbursed medicines in 2021 | 166 632 738 | 918 818 989 | 2 011 294 637 | 639 740 140 | 193 265 817 | 1 872 288 630 | 265 347 153 | 601 078 981 | 101 460 074 | PPP values not available | 210 742 111 | 3 121 591 781 | N/A | 2 247 581 108 | 2 081 347 423 |
| Public spending on the reimbursement of all medicines for hypertension in 2021 | 29 862 931 | 50 226 743 | 127 464 811 | 71 434 829 | 12 470 091 | 164 763 552 | 22 354 180 | 64 401 319 | 15 779 321 | PPP values not available | N/A | N/A | N/A | 241 824 812 | 124 034 134 |
| Public spending on all FDCs used in hypertension treatment in 2021 | 12 482 166 | 24 235 371 | 59 127 115 | 16 470 665 | 6 898 342 | 65 388 374 | 12 320 123 | 30 053 949 | N/A | N/A | N/A | N/A | N/A | 71 363 131 | 33 423 233 |
| 2022 | | | | | | | | | | | | | | | |
| Total public spending on reimbursed medicines in 2022 | 176 950 479 | 972 669 066 | 2 016 958 270 | 721 982 835 | 192 657 628 | 1 828 027 465 | 280 225 035 | 640 253 847 | 97 120 728 | PPP values not available | 203 121 369 | 2 961 989 012 | N/A | 2 604 802 705 | 2 142 174 900 |
| Public spending on the reimbursement of all medicines for hypertension in 2022 | 32 072 440 | 46 301 166 | 124 096 496 | 66 087 177 | 11 658 794 | 157 972 505 | 21 190 032 | 57 179 903 | 14 598 120 | PPP values not available | N/A | N/A | N/A | 262 719 849 | 126 929 173 |
| Public spending on all FDCs used in hypertension treatment in 2022 | 15 466 497 | 22 797 007 | 59 173 918 | 15 455 038 | 7 936 635 | 63 971 472 | 11 767 885 | 27 918 826 | N/A | N/A | N/A | N/A | N/A | 81 521 340 | 34 512 503 |
| 2023 | | | | | | | | | | | | | | | |
| Total public spending on reimbursed medicines in 2023 | N/A | 1 116 473 307 | N/A | 891 760 044 | 210 489 660 | 1 951 986 475 | 315 980 337 | 708 696 294 | 99 323 708 | PPP values not available | N/A | 3 539 584 352 | N/A | N/A | 2 349 428 128 |
| Public spending on the reimbursement of all medicines for hypertension in 2023 | 35 163 835 | 45 059 026 | N/A | 65 649 486 | 11 431 214 | 150 478 197 | 20 954 501 | 54 564 479 | N/A | PPP values not available | N/A | N/A | N/A | N/A | 131 930 964 |
| Public spending on all FDCs used in hypertension treatment in 2023 | 18 329 578 | 22 417 824 | N/A | 15 434 170 | 7 583 278 | 62 615 586 | 11 819 848 | 27 218 046 | N/A | PPP values not available | N/A | N/A | N/A | N/A | 36 011 289 |

* The value provided is for the total distribution of medicines, encompassing both reimbursed and out-of-pocket expenses, for prescription medicines.

N/A - Data not available

Supplementary Table 6. Information about experts.

| Expert | Country | Institution | Role/ Responsibility |
| --- | --- | --- | --- |
| Iris Hoxha | Albania | Faculty of Medicine, Medical University of Tirana, Albania | Faculty Member |
| Alexandra Savova | Bulgaria | --Department "Organization and Economics of Pharmacy", Faculty of Pharmacy, Medical University of Sofia, Bulgaria  --National Council on Prices and Reimbursement of Medicinal Products, Sofia, Bulgaria | Associate professor  Member |
| Pero Draganić | Croatia | --Agency for Medicinal Products and Medical Devices of Croatia, Zagreb, Croatia  --Faculty of Biotechnology and Drug Development, University of Rijeka, Rijeka, Croatia | Principal Advisor for Distribution of Medicines and Pharmacoeconomics  Affiliate |
| Jana Skoupa | Czech Republic | EconHealth s.r.o. Praha, Czech Republic | Managing Director |
| Agnes Männik | Estonia | Institute of Family Medicine and Public Health, University of Tartu, Tartu, Estonia | Analyst |
| Zoltán Kaló | Hungary | --Center for Health Technology Assessment, Semmelweis University, Budapest, Hungary  --Syreon Research Institute, Budapest, Hungary | Professor  Founder, leader |
| Arturs Praskilevics | Latvia | --Department of Pharmaceutical Chemistry, Faculty of Pharmacy, Riga Stradins University, Riga, Latvia  --Red Cross Medical College of Riga Stradins University, Riga, Latvia | Project leader  Director of the "Pharmaceuticals" Study Programme |
| Kristina Garuoliene | Lithuania | Pharmacy and Pharmacology Center, Institute of Biomedical Sciences, Faculty of Medicine, Vilnius University, Vilnius, Lithuania | Associate professor |
| Ljubica Suturkova | North Macedonia | Faculty of Pharmacy, Ss. Cyril and Methodius University in Skopje, Skopje, North Macedonia | Professor |
| Elena Chitan | Republic of Moldova | Department of Social pharmacy, Nicolae Testemitanu State University of Medicine and Pharmacy, Chișinău, Republic of Moldova | Assistant professor |
| Natasa Duborija-Kovacevic | Montenegro | Medical faculty of the University of Montenegro, Department for Pharmacology and Clinical pharmacology, Podgorica, Montenegro | Professor |
| Michał Owca | Poland | Department of Nutrition and Drug Research, Institute of Public Health, Faculty of Health Science, Jagiellonian University Medical College, Kraków, Poland | Assistant |
| Ioana Bianchi-Manaila | Romania | Romanian Association of International Medicine Manufacturers (ARPIM), Bucharest, Romania | External Relations Director |
| Nikola Stefanovic | Serbia | Department of Pharmacy, Faculty of Medicine, University of Nis, Nis, Serbia | Associate Professor |
| Tomas Tesar | Slovakia | Department of Organisation and Management in Pharmacy, Faculty of Pharmacy, Comenius University in Bratislava, Slovakia | Professor |
| Tamás Ágh | Hungary | Medication Adherence Research Group, Center for Health Technology Assessment and Pharmacoeconomic Research, University of Pécs, Pécs, Hungary | Head |

## 1.2 Supplementary Figures

**Supplementary Figure 1.** The number of FDCs reimbursed in a country in relation to the requirements of HTA in the reimbursement process of FDCs containing previously reimbursed components.

**Supplementary Figure 2.** The number of FDCs reimbursed in a country in relation to the time from application to the inclusion in the reimbursement list.
