# Supplementary material for: Availability and reimbursement of fixed-dose combination medicines for hypertension treatment in Central and Eastern Europe
Source: Front Pharmacol. 2026 Oct 8;17:1889485. doi: 10.3389/fphar.2026.1889485 (PMC13687808; doi:10.3389/fphar.2026.1889485)
Supplement: Supplementary file 2 [file Table2.docx]

Fixed-dose combination medicines used in the treatment of arterial hypertension in Europe: availability and reimbursement policy

Introduction

Thank you for your participation. Your expertise and insight are important to our research, and we greatly appreciate your time and contribution.

The study aims to compare the policies of European countries on the reimbursement and pricing of **fixed-dose combination (FDC) medicines** used in the treatment of arterial hypertension. Information on the availability of these FDCs to patients will also be collected.

FDCs used in hypertension treatment are medicines that contain two or more active substances in a tablet or capsule. They are usually sold by pharmacies on prescription (outpatient setting).

For the purpose of this research you will be providing data for your country and as we need to implement fully objective data, we encourage you to consult any other expert from your country, where necessary (e.g., a hypertension specialist, decision maker, an academic).

Please provide your answers (in English) in white-filled spaces. We encourage you to provide additional information in the comment section below each question.

Personal Information

| Please provide information about yourself. | |
| --- | --- |
| Country: |  |
| Full name: |  |
| Conflict of interest (FDCs used in hypertension treatment) |  |
| Affiliation (to be used in the manuscript): |  |
| E-mail address (to be used in the manuscript): |  |
| ORCID ID (to be used in the manuscript): |  |

Your participation in the Research is voluntary and you may refuse to participate or withdraw from it at any time without giving a reason.

| Question . Which of the following best describes your role? | |
| --- | --- |
| ☐ Involvement in the regulatory / legislation process  ☐ Involvement in the reimbursement process  ☐ Evaluation and pricing of medicines  ☐ Pharmacy and/or therapy role  ☐ Education and/or research (academia) | |
| Your comment: |  |

Reimbursement and Pricing

| Question . Are there any official regulations on the reimbursement of FDCs used in hypertension treatment (or FDCs regardless of indication) in your country?  You can select more than one answer. | |
| --- | --- |
| ☐ No  ☐ Yes (in reference to the price of an FDC, e.g., maximum price, price calculation mechanism)  ☐ Yes (in reference to a reimbursement mechanism, e.g., limit group, level of patient co-payment, maximum public payer contribution)  ☐ Yes (specific for FDC application for reimbursement)  ☐ Yes, other (please specify in the comment section below). | |
| Your comment: |  |

| Question . Indicate the aspects of the reimbursement of FDCs that are practically different from those of single-component medicines in your country.  You can select more than one answer. | |
| --- | --- |
| ☐ There are no differences; an FDC is subject to the same reimbursement procedure as the next equivalent of the reimbursed medicine (e.g., generic medicine).  ☐ There are no differences; an FDC is subject to the same procedure as any new medicine that has no reimbursed equivalents (i.e., medicines with the same active component).  ☐ An application for reimbursement is different (please provide more details in the comment section below).  ☐ A pricing procedure is different (please provide more details in the comment section below).  ☐ An assessment prior to reimbursement (i.e., any requirement for reimbursement, health technology assessment) is different (please provide more details in the comment section below).  ☐ Third-party payer* contribution (e.g., maximum contribution) is different or differently estimated (please provide more details in the comment section below).  ☐ Patient co-payment is different or differently estimated (please provide more details in the comment section below).  ☐ Other differences (please specify in the comment section below). | |
| Your comment: |  |

** National health system; government and/or compulsory contributory health care financing scheme*

| Question . Is a formal reimbursement application required for FDCs used in hypertension treatment in your country in order to be refunded by a third-party payer*?  You can select more than one answer. | |
| --- | --- |
| ☐ No  ☐ Yes, always  ☐ It depends on the reimbursement status of FDC components (please specify in the comment section below).  ☐ It depends on other criteria (please describe these criteria in the comment section below). | |
| Your comment: |  |

** National health system; government and/or compulsory contributory health care financing scheme*

| Question . Does the reimbursement application for FDCs used in hypertension treatment include health technology assessment (HTA) dossier or its component(s)?  You can select more than one answer. | |
| --- | --- |
| ☐ N/A; the application is not required.  ☐ No, the HTA dossier is not required.  ☐ Yes, the HTA dossier is always required.  ☐ It depends on the suggested price of an FDC (please provide more details in the comment section below).  ☐ It depends on the reimbursement status of FDC components (please provide more details in the comment section below).  ☐ It depends on other criteria (please describe these criteria in the comment section below). | |
| Your comment: |  |

| Question . Which of the components of the HTA dossier (if any) are required in the application for the reimbursement of FDCs used in hypertension treatment in your country?  You can select more than one answer. If you select more than one answer, please provide details in the comment section below. | |
| --- | --- |
| ☐ N/A; the application or the HTA dossier is not required.  ☐ Clinical analysis (i.e., a systematic review of all clinical evidence on an FDC)  ☐ Simple cost comparison (e.g., the average FDC cost vs the average cost of its components in separate products)  ☐ Full economic analysis (e.g., cost-minimalization analysis, cost-utility analysis)  ☐ Target population size calculation and/or budget-impact analysis | |
| Your comment: |  |

| Question . What level of clinical evidence (if any) is required in the application for the reimbursement of FDCs used in hypertension treatment in your country?  You can select more than one answer. If you select more than one answer, please provide more details in the comment section below. | |
| --- | --- |
| ☐ N/A; the application or the assessment of clinical data is not required.  ☐ The same as for medicine registration (e.g., bioavailability studies); no systematic review is required.  ☐ Systematic review of relevant clinical trials and/or other studies  ☐ It depends on the suggested price of FDC (please provide more details in the comment section below).  ☐ It depends on the reimbursement status of FDC components (please provide more details in the comment section below).  ☐ It depends on other criteria (please describe these criteria in the comment section below). | |
| Your comment: |  |

| Question . If HTA (or at least a simple cost comparison with other medical technologies) is performed as part of the reimbursement process, what are the comparator treatments (“comparators”) for the new FDCs used in hypertension treatment?  You can select more than one answer. If you select more than one answer, please provide more details in the comment section below. | |
| --- | --- |
| ☐ N/A; the application, HTA dossier, or clinical comparison with other medical technologies is not required.  ☐ Components of an FDC in separate products  ☐ Other reimbursed FDCs containing active substances from the same drug classes  ☐ Other reimbursed FDCs containing active substances (no limitation on the drug class)  ☐ Other combinations of medicines in separate products (from the same drug classes)  ☐ Other combinations of medicines in separate products (no limitation on the drug class)  ☐ It depends (please indicate which criteria and comparators are used in the comment section below). | |
| Your comment: |  |

| Question . What type of economic analysis is performed in your country within an HTA dossier for new FDCs used in hypertension treatment?  You can select more than one answer. If you select more than one answer, please provide more details in the comment section below. | |
| --- | --- |
| ☐ N/A; the application or economic analysis is not required.  ☐ Cost-minimalization analysis  ☐ Cost-effectiveness analysis  ☐ Cost-utility analysis  ☐ Other analysis (please specify in the comment section below).  ☐ It depends (please indicate which criteria and analysis are used in the comment section below). | |
| Your comment: |  |

| Question . There is evidence for better adherence to and persistence with FDC treatment versus treatment with FDC components used in separate products. Are these aspects considered in the reimbursement process for new FDCs used in hypertension treatment in your country?  You can select more than one answer. If you select more than one answer, please provide more details in the comment section below. | |
| --- | --- |
| ☐ N/A; the application or HTA dossier is not required.  ☐ No, these aspects are not considered.  ☐ Yes, in the assessment of the clinical value of an FDC  ☐ Yes, in the assessment of the economic value of an FDC (e.g., in economic analysis)  ☐ Yes, in the budget-impact analysis of an FDC  ☐ Yes, in other way (please specify in the comment section below).  ☐ It depends (please indicate which criteria and analysis are used in the comment section below). | |
| Your comment: |  |

| Question . Are clinical experts involved in making the reimbursement decision for FDCs used in hypertension treatment?  You can select more than one answer. If you select more than one answer, please provide details in the comment section below. | |
| --- | --- |
| ☐ No, they are not involved in the reimbursement process.  ☐ Yes, they can formally support the application for reimbursement.  ☐ Yes, their opinion is considered during decision-making.  ☐ Other (please specify in the comment section below). | |
| Your comment: |  |

| Question . Is price negotiation part of the reimbursement process for FDCs used in hypertension treatment in your country?  You can select more than one answer. | |
| --- | --- |
| ☐ N/A; the reimbursement process is not required.  ☐ No, price negotiations are not conducted.  ☐ Yes, price negotiations are always conducted.  ☐ It depends on the suggested price of an FDC (please provide more details in the comment section below).  ☐ It depends on the reimbursement status of FDC components (please provide more details in the comment section below).  ☐ It depends on other criteria (please describe these criteria in the comment section below). | |
| Your comment: |  |

| Question . How long does it take from the application for reimbursement to the formal inclusion of an FDC used in hypertension treatment in the list of reimbursed medicines in your country?  You can select more than one answer. If you select more than one answer, please provide more details in the comment section below. | |
| --- | --- |
| ☐ N/A; the inclusion in the reimbursement list is not required.  ☐ Less than 3 months  ☐ From 3 to 5 months  ☐ From 6 to 8 months  ☐ From 9 to 12 months  ☐ More than 12 months | |
| Your comment: |  |

| Question . Are there any differences in the calculation of the list prices of FDCs used in hypertension treatment in comparison with single-component medicines in your country?  You can select more than one answer. | |
| --- | --- |
| ☐ N/A; the inclusion in the reimbursement list is not required.  ☐ No, there is no difference.  ☐ Yes, there are rules for the **ex-factory price** calculation for an FDC.  ☐ Yes, the **value added tax** **(VAT)** is different (please describe the difference in the comment section below).  ☐ Yes, the **wholesaler** **margin** is different (please describe the difference in the comment section below).  ☐ Yes, the **retail margin** is different (please describe the difference in the comment section below).  ☐ Yes, **other margins or statutory taxes** are different (please describe these margins or taxes and how they are different in the comment section below).  ☐ It depends (please indicate below which criteria are considered and describe their implications). | |
| Your comment: |  |

| Question . Provide detailed information on all taxes and margins used in your country for the calculation of the total official price (including all taxes and margins; usually a retail price) of FDCs used in hypertension treatment in your country. | |
| --- | --- |
| Value-added tax (VAT): |  |
| Wholesaler margin: |  |
| Retail margin: |  |
| Other taxes, margins, and statutory discounts: |  |

| Question . Indicate the character of the list prices of reimbursed FDCs used in hypertension treatment in your country. | |
| --- | --- |
| ☐ N/A; the inclusion in the reimbursement list is not required.  ☐ Fixed prices (all reimbursed FDCs must be sold at a fixed official price in line with the list price).  ☐ Maximum prices (all reimbursed FDCs must be sold at a price not exceeding the price on the list price).  ☐ It depends (please indicate below which criteria are considered and describe their implications). | |
| Your comment: |  |

| Question . Are there any limits other than the list prices (e.g., maximum public payer contribution) for reimbursed FDCs used in hypertension treatment in your country?  You can select more than one answer. If you select more than one answer, please provide details in the comment section below. | |
| --- | --- |
| ☐ No  ☐ Yes, there are limit groups with shared limit for similar medicines (e.g., the same active substance, similar efficacy).  ☐ Yes, the limit is established individually for each drug.  ☐ It depends (please indicate below which criteria are considered and describe their implications). | |
| Your comment: |  |

| Question . How does the reimbursement limit impact patient payment (co-payment) for FDCs used in hypertension treatment in your country?  You can select more than one answer. | |
| --- | --- |
| ☐ N/A; there is no limit.  ☐ The patient can buy the medicine at a higher price than the limit but pays the difference between the price and the limit.  ☐ The patient can buy the medicine at a higher price than the limit but pays 100% of the price (no reimbursement if the price is higher than the limit).  ☐ The patient can buy the medicine at a higher price than the limit and the medicine is reimbursed, but individual consent from the third-party payer is required.  ☐ Other (please indicate below which criteria are considered and describe their implications). | |
| Your comment: |  |

| Question . Choose the sentence (or sentences) that best describes the ex-factory list price of FDCs used in hypertension treatment in your country.  You can select more than one answer. If you select more than one answer, please provide details in the comment section below. Please assume that the active components of FDCs are reimbursed. | |
| --- | --- |
| *The* ***ex-factory*** *list price (without VAT or any margin) of a reimbursed FDC used in hypertension treatment*  ☐ … is estimated from the price of **all its components** (adjusted for the number of mg of each component).  ☐ … is estimated from the price of **only one of its components** (adjusted for the number of mg of this component).  ☐ … is independent of the list prices of their components.  ☐ It depends (please indicate below which criteria are considered and describe their implications).  ☐ N/A; the inclusion in the reimbursement list is not required or there are no FDC reimbursed. | |
| Your comment: |  |

| Question . Indicate the relation of the ex-factory price of reimbursed FDCs and the prices of reimbursed components in your country.  Select an appropriate option from the drop-down menu. You can indicate more than one relation. Please assume that the active components of FDCs are reimbursed. | |
| --- | --- |
| The FDC price is *Choose an item.* as / than the *Choose an item.* (adjusted for the number of mg of each component).  The FDC price is *Choose an item.* as / than the *Choose an item.* (adjusted for the number of mg of each component).  The FDC price is *Choose an item.* as / than the *Choose an item.* (adjusted for the number of mg of each component). | |
| Your comment: |  |

| Question . Choose the sentence that best describes the limit of reimbursement (i.e., the amount of third-party payer’s contribution) of FDCs used in hypertension treatment in your country.  You can select more than one answer. If you select more than one answer, please provide details in the comment section below. | |
| --- | --- |
| *The list maximum amount of reimbursement (e.g., maximum public payer’s contribution) of a reimbursed FDC used in hypertension treatment*  ☐ … is estimated from the reimbursement amount/limit for **all of its components** (adjusted for the number of mg of each component).  ☐ … is estimated from the reimbursement amount/limit for **only one of its components** (adjusted for the number of mg of this component).  ☐ … is independent of the reimbursement amount/limit for its components.  ☐ It depends (please indicate below which criteria are considered and describe their implications).  ☐ N/A; the inclusion in reimbursement list is not required or there are no FDCs reimbursed. | |
| Your comment: |  |

| Question . Choose the sentence that best describes patient co-payment for reimbursed FDCs used in hypertension treatment in your country.  You can select more than one answer. If you select more than one answer, please provide details in the comment section below. | |
| --- | --- |
| *The amount of patient co-payment for a reimbursed FDC used in hypertension treatment*  ☐ … is estimated from the amount of patient co-payment for **all its components** (adjusted for the number of mg of each component)  ☐ … is estimated from the amount of patient co-payment for **only one of its components** (adjusted for the number of mg of this component)  ☐ … is independent of the amount of patient co-payment for its components  ☐ It depends (please indicate below which criteria are considered and describe their implications).  ☐ N/A; the inclusion in reimbursement list is not required or there are no FDCs reimbursed. | |
| Your comment: |  |

| Question . Does the level of patient co-payment (e.g., percentage of the price) for FDCs used in hypertension treatment differ from the level of patient co-payment for single-component medicines?  You can select more than one answer. | |
| --- | --- |
| ☐ No, the level of patient co-payment does not depend on the number of active substances.  ☐ Yes, it differs from the level of patient co-payment for single-component medicines (please indicate the differences in the comment section below).  ☐ It depends (please indicate below which criteria are considered and describe their implications). | |
| Your comment: |  |

| Question . What aspects determine the patient co-payment for reimbursed medicines in your country? | |
| --- | --- |
| ☐ Medicine’s therapeutic indication (e.g., disease prevalence or importance of the disease)  ☐ Patient’s income  ☐ Duration of medicine use (e.g., short-term use vs long-term, chronic use)  ☐ Price of the medicine  ☐ Total amount of the patient co-payment for reimbursed medicines  ☐ Insurance plan  ☐ Type or number of active substances in the medicine  ☐ Other (please specify in the comment section below) | |
| Your comment: |  |

Question . Assume that there is a theoretical FDC containing two active substances or components (X and Y).

The ex-factory price suggested by the manufacturer is €70 for 30 tablets of 10 mg of X and 50 mg of Y.

Both components of the FDC (X and Y) are reimbursed in your country (as separate single-component products).

The average price (without VAT, margins, and taxes) of the reimbursed component X (30 tablets of 10 mg of X) is €15 (range: €10 to €20), and the average price (without VAT, margins and taxes) of the reimbursed component Y (30 tablets of 50 mg of Y) is €30 (range: €20 to €40).

Please share your opinion at what price will the FDC be most likely reimbursed in your country?

| Question . How often does the list of reimbursed medicines change in your country?  Please select only one answer. | |
| --- | --- |
| ☐ Very frequently (every month or every two months)  ☐ Frequently (every three or four months)  ☐ Rarely (once or twice per year)  ☐ Occasionally (less than once a year) | |
| Your comment: |  |

FDC Availability

| Question . How do you rate the availability of FDCs used in hypertension treatment in your country?  Please select only one answer. | |
| --- | --- |
| ☐ High (most FDCs are reimbursed, patient co-payment is not higher than that for using FDC components in separate products, and there are no specific restrictions on using FDCs).  ☐ Moderate (some FDCs are reimbursed and/or patient co-payment is higher than that for using FDC components in separate products).  ☐ Low (few or no FDCs are reimbursed, and/or patient co-payment is much higher than that for using FDC components in separate products, and/or there are restrictions on using FDCs).  ☐ Not sure/I do not know. | |
| Indicate any limitations of the reimbursement process or health care barriers that can reduce the availability of FDCs used in hypertension treatment in your country: |  |

| Question . Are international guidelines on hypertension treatment implemented in your country (especially in relation to FDCs used in hypertension treatment)? | |
| --- | --- |
| ☐ No  ☐ Yes  ☐ Partially (please specify in the comment section below) | |
| Your comment: |  |

| Question . Please select FDCs used in hypertension treatment that are reimbursed in your country and contain two groups of active substances. Provide a list of unique combinations of the active substances and their doses within these FDCs.  The groups considered:   - angiotensin receptor blockers (ARB): azilsartan, candesartan, eprosartan, fimasartan, irbesartan, losartan, olmesartan, saprisartan, telmisartan, valsartan - angiotensin‐converting enzyme inhibitors (ACEI): alacepril, benazepril, captopril, ceronapril, cilazapril, enalapril, fosinopril, imidapril, lisinopril, perindopril, quinapril, ramipril, trandolapril, zofenopril - calcium channel blockers (CCB): amlodipine, aranidipine, azelnidipine, barnidipine, benidipine, cilnidipine, clevidipine, efonidipine, felodipine, isradipine, lacidipine, lercanidipine, manidipine, nicardipine, nifedipine, nilvadipine, nimodipine, nisoldipine, nitrendipine, pranidipine - thiazide and thiazide‐like diuretics (DIU): bendroflumethiazide, chlorothiazide, chlortalidone, cyclopenthiazide, hydrochlorothiazide, indapamide, methyclothiazide, metolazone - beta-blockers  (BB): acebutolol, atenolol, betaxolol, bisoprolol, bucindolol, butaxamine, carteolol, carvedilol, celiprolol, esmolol, labetalol, metoprolol, nadolol, nebivolol, oxprenolol, penbutolol, pindolol, propranolol, sotalol, timolol | |
| --- | --- |
| **Reimbursed FDC (groups of active substances)** | **All reimbursed combinations of active substances and their doses in an FDC**  *e.g., Amlodipine 10 mg + Valsartan 160 mg (in a tablet or capsule)* |
| ☐ ARB + CCB |  |
| ☐ ACEI + DIU |  |
| ☐ ARB + DIU |  |
| ☐ ARB + ACEI |  |
| ☐ ACEI + CCB |  |
| ☐ CCB + DIU |  |
| ☐ ARB + BB |  |
| ☐ ACEI + BB |  |
| ☐ CCB + BB |  |
| ☐ DIU + BB |  |
| Other FDCs with **two** **active substances** (not listed above, e.g., statin + CCB or ARB)  Please indicate active substances and their doses in a tablet or capsule, e.g., Amlodipine 10 mg + Valsartan 160 mg | |
|  | |

Question . Please provide a list of reimbursed FDCs used in hypertension treatment in your country and containing three or more active substances.

Please indicate active substances and their doses in a tablet or capsule, e.g., Amlodipine 10 mg + Valsartan 160 mg + Hydrochlorothiazide 12,5 mg

| Question . Please provide information (in your country currency) on:   - total public spending on reimbursed medicines, - public spending on the reimbursement of all medicines for hypertension, and - public spending on all FDCs used in hypertension treatment   in your country, in the years 2021-2023.  Please provide an exact or estimated amount. | | | |
| --- | --- | --- | --- |
|  | **All medicines** | **All medicines for hypertension** | **FDCs for hypertension** |
| **2021** (in local currency) |  |  |  |
| **2022** (in local currency) |  |  |  |
| **2023** (in local currency) |  |  |  |
| Source (to be included in the manuscript) |  | | |

Question .

Please provide a link to a website or file that contains a list of reimbursement medicines in your country (list prices of reimbursed medicines, including FDCs, patient co-payment, patient price per package, the year of obtaining the reimbursement status for a given FDC, etc.).

Optionally, you can send the file containing the list via email.
